# Micro- and Nanoplastics as Potential Drivers of Dilated Cardiomyopathy

**DOI:** 10.3390/life16060916

**Published:** 2026-05-29

**Authors:** Joshua Xu, Sanjay Sivalokanthan

**Affiliations:** 1 Faculty of Medicine, Imperial College London, London SW7 2AZ, UK; joshua.xu19@imperial.ac.uk; 2 Mount Sinai Fuster Heart Hospital, Icahn School of Medicine, New York, NY 10025, USA

**Keywords:** microplastics, nanoplastics, myocardium, cardiomyopathy, oxidative stress, environmental health, exposome

## Abstract

Dilated cardiomyopathy (DCM) is a leading cause of heart failure, but up to 50% of cases have no definitive etiology. Genetic susceptibility alone does not account for phenotypic inconsistency, so a ‘two-hit’ model has been proposed to explore the spectrum of gene-environment interactions. Certain triggers, such as alcohol, chemotherapy agents, and viral myocarditis, are well-established second hits in the pathogenesis of DCM. The exposome, which encompasses environmental and social exposures across the lifespan, provides a more comprehensive framework to understand these interactions. In patients with DCM, air pollution and heavy metals have already been associated with higher rates of mortality and heart failure hospitalization. Microplastics and nanoplastics (MNPs) are novel components of the exposome. They form from the degradation of plastics and enter the circulatory system primarily through ingestion and inhalation. They have recently been found in human cardiovascular tissue, including atherosclerotic plaques and the myocardium. In vivo and in vitro models consistently demonstrate that MNPs induce oxidative stress, mitochondrial dysfunction, and calcium dysregulation. These pathways are shared with established cardiotoxins and converge on cardiomyocyte death, fibrosis, and eccentric ventricular remodeling, which is consistent with the pathogenesis and phenotype of DCM. In genetically susceptible individuals, MNP exposure may therefore contribute to the progression from subclinical myocardial injury to overt systolic dysfunction. This narrative review synthesizes preclinical mechanistic evidence linking MNP exposure to myocardial injury, compares the underlying mechanisms with those of other environmental pollutants and cardiovascular toxins, and integrates these findings within the proposed ‘two-hit’ model of DCM. Whether MNP exposure contributes to DCM in humans remains to be established, but understanding the potential consequences of MNPs has important implications for prevention, therapeutic development and health policy. Standardization of detection methods, chronic low-dose exposure models, and prospective human studies using functional cardiac assessment are needed before translating these experimental findings into clinical practice.

## 1. Introduction

Heart failure remains a leading cause of morbidity and mortality, affecting an estimated 55.5 million people worldwide [1]. Despite major advancements in treatment, the overall prognosis remains poor, and increasing attention is now being paid towards prevention. Cardiomyopathy represents a heterogeneous group of myocardial disorders contributing to this global burden and is characterized by structural and functional abnormalities of the myocardium. Among this group, dilated cardiomyopathy (DCM) remains poorly characterized and understood [2]. DCM is defined by dilation of the left ventricle and systolic dysfunction not caused by abnormal loading conditions or myocardial ischemia. Up to 50% of patients remain without a definitive etiology even after genetic testing and biopsy assessments [3]. This lack of certainty is likely due to the complex gene-environment interactions. Genetic variants such as TTNtv exhibit incomplete penetrance, supporting a ‘two-hit hypothesis’ in which genetic susceptibility lowers the threshold for phenotypic expression following exposure to environmental stressors, including alcohol, viral myocarditis, and anthracyclines [4]. Direct evidence for this framework was provided by a retrospective longitudinal cohort study of 105 genotyped individuals from families with DCM-causing variants, which found that DCM-promoting environmental factors were significantly enriched in genotype-positive, phenotype-positive individuals and were associated with a twofold increased risk of DCM onset [5]. In addition, 78% of left ventricular ejection fraction (LVEF) fluctuation episodes were due to environmental factors, and TTNtv carriers showed near-uniform susceptibility to these exposures, suggesting they may represent a particularly vulnerable group to environmental toxins [5].

Increasing evidence indicates that environmental exposures affect the development, progression, and outcomes of heart failure (HF). Whilst research has traditionally focused on genetic predisposition and conventional risk factors, emerging evidence is beginning to uncover the role of cumulative environmental exposure as a major contributor to cardiovascular health across the lifespan [6,7,8].

The exposome encompasses external environmental, lifestyle, and socioeconomic factors, providing a holistic framework for disease [9]. Certain pollutants have already been associated with both worse outcomes in patients with DCM and heart failure hospitalizations. For example, fine particulate matter < 2.5 μm (PM_2.5_) has been associated with adverse cardiac remodeling, and heavy metals such as cadmium have been associated with impaired myocardial contractility [10,11]. At a cellular level, chronic exposure to these toxins induces oxidative stress, inflammation, and endothelial injury [11,12]. These findings suggest that environmental exposures may contribute to the development and exacerbation of cardiovascular disease.

Following recent advancements in detection techniques, plastic has emerged as a novel component of this framework. Global plastic production has grown more than 250-fold from 1950 to 2022, and without appropriate interventions, levels are projected to triple by 2060 [13]. After plastics are released into the environment, they degrade to form microplastics (particles < 5 mm) and nanoplastics (particles < 1 µm) (MNPs) [14]. They slowly break down in landfills and aquatic environments, contaminating ecosystems and entering the human food and water systems. Following exposure, they deposit in tissues including the lung, placenta, liver, and, more recently, within atherosclerotic plaques and the myocardium [15,16,17]. Whether this deposition within cardiovascular tissue carries functional consequences is still being explored and has prompted global initiatives such as the “Lancet Countdown on Health and Plastics’’ to monitor exposure and inform policy [18].

Oxidative stress, mitochondrial and endoplasmic reticulum dysregulation play central roles in cardiomyocyte injury and the pathogenesis of toxic dilated cardiomyopathy [19]. Recent preclinical studies have found that MNPs activate these same pathways, mechanisms shared with anthracycline- and alcohol-induced cardiomyopathy, raising the possibility that MNPs may represent a previously unrecognized environmental second hit. However, direct evidence linking MNP exposure to a DCM phenotype remains limited, and this review aims to address this gap.

In this review, we synthesize the current preclinical evidence and propose a mechanistic framework linking MNP exposure with DCM. We focus specifically on oxidative stress, mitochondrial dysfunction, and calcium dysregulation as the primary pathways driving cardiomyocyte injury and explore their role in the gene-environment context of the two-hit hypothesis. We aim to provide a perspective on how these environmental pollutants may contribute to the global burden of heart failure and identify key gaps that future research should address.

## 2. Methods

This narrative review was conducted by searching PubMed, MEDLINE, and Embase from inception to March 2026. Search terms included combinations of microplastics, nanoplastics, dilated cardiomyopathy, cardiomyopathy, myocardial injury, oxidative stress, mitochondrial dysfunction, calcium dysregulation, ferroptosis, pyroptosis, apoptosis, fibrosis, exposome, and related terms. Studies were selected based on their relevance to MNP-induced cardiovascular and myocardial injury, with no restriction on study design or model used. Given the contemporary nature of this area of research, a narrative rather than systematic approach was used to allow the integration of mechanistic, preclinical, and translational evidence across multiple disciplines.

## 3. Background

### 3.1. Introduction to MNPs

MNPs primarily arise from the breakdown of larger plastic items through physical, chemical, or biological processes. Microplastics (MPs) and nanoplastics (NPs) are particles defined as <5 mm and <1 µm in size, respectively. They have a range of shapes, including spherical beads, fibers, or granules with broken or irregular edges, factors which can affect their biological interactions [20]. Their physicochemical properties, such as charge and hydrophobicity, can further influence cellular uptake, tissue accumulation, and the adsorption of other contaminants [21].

MNPs can be further classified by polymer composition, with common types including polystyrene (PS), polypropylene (PP), polyethylene terephthalate (PET), polyethylene (PE), polyvinylchloride (PVC), and polymethylmethacrylate (PMMA). Of these, PVC has demonstrated the greatest cytotoxicity at equivalent particle size and dose [22]. MNPs may also act as vectors for harmful agents, including heavy metals, plasticizers, and pathogens, thereby further amplifying their toxic effects [23,24,25]. Unlike other toxins, such as alcohol, which can be metabolized, MNPs can persist in tissues indefinitely, potentially with more permanent consequences.

### 3.2. Routes of Exposure

Ingestion, inhalation, and dermal contact are the main routes of human exposure to MNP, with ingestion being the most common [26]. MNPs have been detected in food and water sources, with higher concentrations in seafood due to bioaccumulation [27]. Following ingestion, larger particles are excreted, but smaller nanoplastics can cross the epithelium and enter the circulatory system [28]. Inhaled particles may deposit within the respiratory tract, where mucociliary clearance can remove larger particles. However, smaller nanoplastics can reach the alveolar spaces and enter systemic circulation [29]. Dermal exposure is considerably less significant, as the skin can act as an effective barrier against most particles.

### 3.3. Cardiovascular Distribution and Clinical Associations

Once in the systemic circulation, MNPs can reach tissues throughout the body. The concentration of plastic particles in the blood of healthy volunteers has been measured at 1.6 μg/mL, with the most abundant particles identified as PET, PE, and PS [30]. MNPs have been detected in various human tissues like the lungs, liver, kidneys, and placenta [31]. The cardiovascular system appears to be a particular site of accumulation, as MNPs have been found in vascular tissues, including the saphenous vein and femoral, coronary, and carotid arteries, with higher concentrations in atherosclerotic plaques [32,33]. A landmark observational study found polyethylene in 58% and PVC in 12% of carotid plaques, and microscopy showed MNP fragments within macrophages and the extracellular matrix, accompanied by increased inflammatory cytokines, a phenotype suggestive of a more vulnerable plaque [34]. Over the three-year follow-up period, MNP presence was associated with a four-fold increase in major adverse cardiovascular events [34]. Similar findings were reported in coronary artery samples from patients undergoing coronary angiography, where PVC was associated with a significant increase in major adverse cardiovascular events [35]. These results suggest that MNPs may be involved in atherosclerosis, but whether they are a cause or a result of prior injury remains unanswered. More recently, MNPs have been detected in the myocardium and pericardium of patients undergoing cardiac surgery [36]. Preclinical studies using human induced pluripotent stem cell-derived cardiomyocytes have found a dose-dependent increase in oxidative stress and apoptosis following MNP exposure [37]. Collectively, these findings suggest an association between MNP exposure and adverse cardiovascular outcomes, but mechanistic clarification is required before causation can be established.

### 3.4. Detection Methods

Accurate assessment of the health risks with MNPs depends on rigorous detection, characterization, and quantification methods. Most research currently uses either particle-based or mass-based assessment methods.

Particle-based methods enable the morphological characterization of MNPs. Examples include optical microscopy, which can detect microplastics approximately 1 µm in size and scanning electron microscopy, which offers higher resolution, but neither method is suitable for detecting nanoplastics [38]. Vibrational spectroscopy, which includes Fourier-transform infrared (FTIR) spectroscopy and Raman spectroscopy, is able to characterize compounds based on the vibrations of chemical bonds. Micro-FTIR is better for microplastics, whilst Raman spectroscopy is preferred for nanoplastics; so the two are frequently used together to improve sensitivity and resolution [39]. Mass-based assessment offers higher sensitivity and specificity but is more destructive. Pyrolysis-gas chromatography–mass spectrometry (Py-GCMS) involves the thermal decomposition of polymers for quantitative and qualitative analysis, but it cannot determine the particle morphology [40].

Taken together, particle-based methods provide information on morphology but are limited in detecting smaller nanoplastics, whilst mass-based techniques offer precise quantitative analysis but lack spatial resolution. Hybrid platforms are currently being developed to address these limitations, and developments in these detection techniques may help to reduce variability by standardizing molecular investigation approaches.

## 4. Mechanisms and Functional Consequences of MNP-Induced Myocardial Injury

### 4.1. Cellular Mechanisms

MNPs exert cardiotoxic effects not from a single insult but from a network of dynamic cellular injury pathways. Central to this network is oxidative stress, which acts as both the initial trigger and amplifier of downstream damage. Mitochondrial dysfunction and ER stress are both directly activated by this oxidative stress and are linked to each other through calcium dysregulation. Together, these pathways converge on cardiomyocyte death and fibrosis, which are the structural and functional features of a DCM phenotype.

#### 4.1.1. Oxidative Stress

Oxidative stress is defined as an imbalance between ROS production and the cell’s antioxidant capacity and is a central driver of cardiomyocyte injury and progression of heart failure [41]. ROS are primarily generated by mitochondria and NADPH oxidase (NOX) under pathological conditions, leading to damage to proteins, lipids, DNA, and organelles. A self-perpetuating cycle is often established, in which chronic mitochondrial ROS generation triggers mitochondrial DNA release, impairs the electron transport chain, and further increases ROS production, culminating in cardiomyocyte apoptosis and fibrosis [42]. This cycle may promote pathological hypertrophy, cardiomyocyte loss, and fibrosis through NF-κB and MAPK signaling [43].

Oxidative stress has also been identified as a central mechanism of MNP-driven toxicity across major organ systems [44,45]. Multiple studies across fish, chicken, and rodent models have consistently found that PS-MPs and PS-NPs increase myocardial malondialdehyde (MDA), a marker of lipid peroxidation, whilst reducing antioxidant enzymes like superoxide dismutase (SOD), glutathione peroxidase (GSH-Px), and catalase (CAT) [37,46,47,48,49,50,51,52,53,54,55,56]. The magnitude of oxidative stress is also dependent on particle size, with smaller NPs having greater cellular penetration and producing a more pronounced oxidative stress response than larger MPs [49,50]. PP-MPs and PET-MPs have demonstrated comparable effects, although physicochemical differences between polymer types, particle sizes, and adsorbed contaminants may also modulate the response [57,58].

Upstream of mitochondrial ROS generation, the TLR/NOX2 signaling pathway has been identified as a potential initiating mechanism. TLR4 is a pattern recognition receptor of the innate immune system that activates in response to exogenous danger signals and results in the upregulation of NOX activity and increased ROS production. In cardiac tissue of carp exposed to PS-NPs, both TLR4 and NOX2 were upregulated compared to controls, suggesting that the innate immune activation may amplify the mitochondrial ROS production [49].

Among the downstream consequences of MNP-induced oxidative stress, NF-κB-mediated inflammation and the p38/MAPK pathways are particularly relevant to DCM pathogenesis. The amount of oxidative stress appears to determine the downstream outcome. A high burden of oxidative stress drives apoptosis, whilst intermediate levels preferentially activate NF-κB, promoting adverse remodeling prior to cell death [59]. This dose-dependent outcome may partly explain the spectrum of cardiac phenotypes observed across MNP exposure studies. Within the MAPK family, p38 kinase has been most implicated and consistently linked to negative inotropic effects and impaired myofilament calcium sensitivity [60]. This provides a potential explanation for the link between oxidative stress and contractile dysfunction. NP exposure has been shown to activate the TNF-α/NF-κB and p38/MAPK signaling pathways, with TNF-α exerting independent negative inotropic effects by disrupting intracellular calcium homeostasis [46]. MNP-induced ROS also activates the TGF-β1/Smad2/3 signaling, which is associated with myofibroblast proliferation, as discussed further in Section 4.3 [54].

Overall, NF-κB and p38/MAPK pathways emerge as the primary downstream effectors of MNP-induced oxidative stress, consistent with pathways implicated in heart failure progression and supporting a plausible role for MNPs in DCM pathogenesis [43].

#### 4.1.2. Mitochondrial Dysfunction

Mitochondria serve as the primary energy source of the cardiomyocyte and generate ATP via oxidative phosphorylation to meet its high energy demands, whilst also playing important roles in calcium homeostasis, ROS generation, and cell death signaling, positioning them as a critical point of convergence among cellular injury pathways. Mitochondrial quality is maintained through a balance of fusion and fission through mitophagy, which is the selective autophagy of damaged mitochondria. Disruption of this equilibrium has been consistently implicated in the pathogenesis of heart failure, where dysfunction leads to energy insufficiency, calcium dysregulation, and oxidative stress, all of which contribute to adverse cardiac remodeling [61].

MNPs accumulate within mitochondria and disrupt their dynamics across multiple interconnected pathways [62]. The sequence reproduced consistently across fish, avian, and rodent models involves mitochondrial calcium overload, reduction in mitochondrial membrane potential (MMP), impaired ATP synthesis, and release of cytochrome C into the cytoplasm [55,56,58,63]. Cytochrome C alters the Bax/BCL2 ratio, activating the mitochondrial-mediated apoptosis pathway, as discussed further in Section 4.2. On histological examination, cardiac tissue from MNP-exposed models exhibits corresponding ultrastructural abnormalities, including mitochondrial fragmentation, loss of cristae, and increased vacuolization [64].

Disruption of mitochondrial dynamics may be partly mediated by the AMPK-PGC-1α pathway, a key regulator of cellular energy homeostasis. Impairment of this pathway has been shown to dysregulate mitochondrial fusion and fission factors in PS-MP-exposed cardiomyocytes, thereby impairing the cell’s ability to maintain normal energy homeostasis [53]. However, this finding is derived from a single avian model and requires replication in mammalian systems before firm conclusions can be drawn.

The relationship between mitochondrial dysfunction and calcium dysregulation is bidirectional. MNP-induced impairment of sarcoplasmic reticulum calcium homeostasis results in the pathological transfer of calcium into the mitochondrial matrix, which depolarizes the inner membrane, suppresses ATP production, and generates further ROS, establishing a self-perpetuating cycle of injury [55]. MNPs have also been linked to reduced DKK3 expression, which impairs mitophagy, resulting in the buildup of damaged mitochondria and further contributing to this cycle [65]. In hiPSC-derived cardiomyocytes, this combination of mitochondrial ROS generation, calcium dysregulation, and defective mitophagy was associated with the activation of hypertrophic signaling pathways and contractile dysfunction [37].

A further consequence of severe mitochondrial injury is activation of the cGAS-STING pathway. Mitochondrial calcium overload can trigger the release of mtDNA into the cytoplasm, where it acts as a damage-associated molecular pattern (DAMP) and activates the cGAS-STING signaling [66]. This pathway has been associated with cellular senescence and inflammation. Accumulation of senescent cardiomyocytes and fibroblasts is a recognized feature of advanced DCM [67]. Whilst direct evidence linking cGAS-STING activation to DCM is limited, and its relevance to MNP-induced injury remains speculative, it represents a plausible mechanism by which chronic mitochondrial injury may contribute to myocardial deterioration.

Mitochondrial dysfunction, therefore, occupies a central amplifying role in MNP-induced cardiotoxicity. By converting initial oxidative stress into energetic failure and cell death, it may contribute to progressive cardiomyocyte loss consistent with the DCM phenotype.

#### 4.1.3. Endoplasmic Reticulum Stress and Calcium Dysregulation

The endoplasmic reticulum plays an important role in cardiomyocytes for protein folding, lipid synthesis, and intracellular calcium regulation [68]. Under physiological conditions, the rhythmic rise and fall of cytosolic calcium concentration control the excitation-contraction coupling. This process is primarily controlled by the sarco-endoplasmic reticulum calcium ATPase (SERCA), which is required for the re-uptake of calcium into the SR following each contraction. Reduced SERCA activity, which is a hallmark of heart failure, prolongs cytosolic calcium elevation and reduces contractile function [69]. Disruption of ER homeostasis, most commonly due to ROS, activates the unfolded protein response (UPR), which is initially adaptive but, when chronically activated, may promote apoptosis and remodeling [70].

Emerging evidence suggests that MNPs contribute to ER stress through direct accumulation in the ER and ROS-mediated disruption [71]. The primary functional consequence is inhibition of SERCA activity. Transient measurements of calcium following short- and long-term PS-NP exposure have shown a time-dependent reduction in SERCA function, with decreased calcium transient amplitude and impaired calcium reuptake, associated with prolonged calcium decay [72]. In hiPSC-derived cardiomyocytes, this translated into suppressed contractility [37]. Beyond its effect on the ER, SERCA inhibition may drive pathological calcium transfer into the mitochondrial matrix, potentially triggering mitochondrial dysfunction described in Section 4.1.2, providing a mechanistic link between ER calcium dysregulation, energetic failure, and cardiomyocyte loss.

Another complementary mechanism has been proposed for the dysregulation of calcium homeostasis. PS-NPs may adsorb to the plasma membrane, extracellularly or internally, thereby physically disrupting SR calcium cycling and interfering with excitation-contraction coupling, independent of the ER stress pathway [73]. This hypothesis requires further validation but raises the possibility that MNPs impair calcium homeostasis from both intracellular signaling and direct membrane-level interference.

At the molecular level, MNP-induced ER stress activates the UPR by upregulating CHOP and spliced XBP-1, transcription factors that shift cellular responses towards pro-apoptotic gene expression. CHOP activation is associated with mitochondrial apoptosis, providing a link between ER stress and Bax/BCL2 dysregulation described in Section 4.2.1. UPR activation also impairs functional protein translation, reducing transmembrane ion channel expression and conductance. A single study reported that this was associated with a proarrhythmogenic phenotype with reduced action potential upstroke velocity and plateau [74]. Although arrhythmia is not a core feature of DCM, this finding highlights potential electrophysiological problems that may arise from MNP-induced ER stress.

Intersecting with both ER stress and mitochondrial dysfunction is the impairment of autophagic flux. In AC16 cardiomyocytes, PS-MPs exposure was associated with impaired autophagy, resulting in accumulation of damaged proteins and organelles and reduced cellular viability [75]. Autophagy is an important quality-control mechanism for both misfolded ER proteins and damaged mitochondria, so disruption of this process may amplify injury pathways, accelerating the progression to irreversible cardiomyocyte injury.

**Figure 1:**
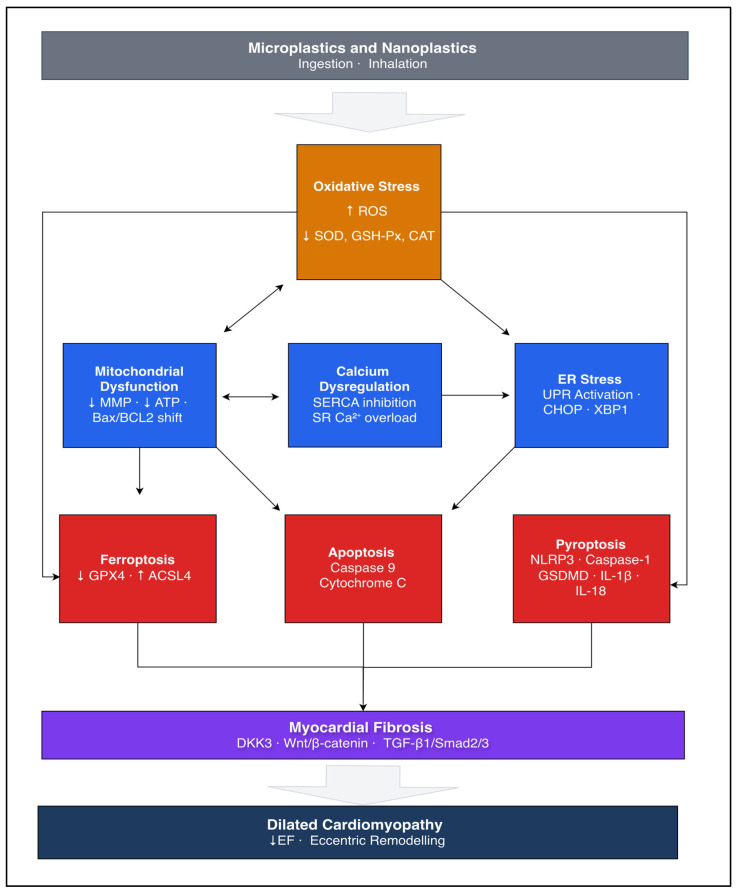
Summary of mechanisms leading to dilated cardiomyopathy.

Oxidative stress, mitochondrial dysfunction, and ER stress, along with calcium dysregulation, form an interconnected network of cellular injury mechanisms. MNP-induced ROS formation is the initiator, impairing mitochondrial membrane integrity and disrupting ER balance. SERCA inhibition may result in excess calcium transfer into the mitochondrial matrix, further reducing the membrane potential and potentially promoting the cell towards apoptosis. ER stress further compounds this pathway through CHOP- and XBP-1-mediated pro-apoptotic signaling and impairs autophagy. In cardiomyocytes with genetic susceptibility or pre-existing damage, the threshold for pathway activation may be lower, consistent with the two-hit model of DCM. The downstream consequences of these pathways include cardiomyocyte death via apoptosis, pyroptosis, and ferroptosis, which are addressed in the following section. The interconnected nature of these pathways and their converge on a DCM phenotype is illustrated in Figure 1.

The figure illustrates how microplastics and nanoplastics (MNPs) enter the systemic circulation primarily through ingestion and inhalation. After depositing in cardiomyocytes, MNPs induce oxidative stress by producing reactive oxygen species (ROS), depleting antioxidant enzymes (SOD, GSH-Px, CAT). Oxidative stress then promotes mitochondrial dysfunction, calcium dysregulation, and endoplasmic reticulum (ER) stress. SERCA inhibition prolongs cytosolic calcium, leading to its transfer into the mitochondrial matrix and further mitochondrial dysfunction and ROS formation. These pathways then converge on apoptosis, pyroptosis, and ferroptosis. Gradual loss of cardiomyocytes activates myofibroblasts via Wnt/beta-catenin, DKK3, and TGF-beta1/smad2/3 pathways, culminating in myocardial fibrosis and eccentric left ventricular hypertrophy, mirroring the dilated cardiomyopathy phenotype. Bidirectional arrows show self-amplifying feedback relationships. Solid arrows indicate direct activation; bidirectional arrows indicate self-amplifying feedback relationships.

### 4.2. Cardiomyocyte Death

Loss of cardiomyocytes is a core and irreversible feature of heart failure progression and represents the terminal consequence of the cellular injury pathways outlined in Section 4.1. MNPs have been implicated in three distinct forms of regulated cell death in preclinical models: apoptosis, pyroptosis, and ferroptosis.

#### 4.2.1. Apoptosis

Apoptosis occurs via two main pathways, the external death receptor pathway and the intrinsic mitochondrial pathway [76,77]. In the myocardium, apoptosis is predominantly mediated by the intrinsic pathway, which is regulated by the balance between the pro-apoptotic Bax and anti-apoptotic BCL2 on the outer mitochondrial membrane. Alterations in this ratio lead to mitochondrial membrane permeabilization, cytochrome C release, and caspase-9 activation. This pathway is highly sensitive to oxidative stress, calcium dysregulation, and inflammatory signaling [76]. Cardiomyocyte apoptosis is recognized as a key driver of the transition from compensatory hypertrophy to contractile dysfunction and heart failure [61].

Multiple experimental studies across fish, rodent, and avian models involving PS-NPs and PP-MPs have consistently shown that MNP-induced injury converges on this pathway through elevated Bax/BCL2 ratios, increased mitochondrial membrane permeabilization, and caspase-9 activation [78,79,80]. These findings align with Section 4.1, which found that ROS generation and mitochondrial calcium overload shift the Bax/BCL2 towards apoptosis, supporting the interpretation that cardiomyocyte death may represent the terminal consequence of a network of pathways rather than an independent event.

TLR4, previously identified in Section 4.1.1 as an upstream regulator of NOX2-mediated ROS, acts as an additional route to apoptosis through NF-κB activation and a pro-apoptotic Bax/BCL2 ratio, a pathway shared with DCM pathogenesis [79,81]. TLR4-deficient mice are protected against viral-induced cardiac injury, which is consistent with a role for TLR4 as a mediator of environmentally triggered apoptosis [82].

The extent of MNP-induced apoptosis is further modulated by the exposure characteristics. Smaller nanoplastics have been shown to result in greater oxidative stress by increasing NOX2 and TLR4 activation [49]. In addition, co-exposure of MNPs with cadmium has been shown to produce greater apoptotic responses than either exposure alone, consistent with the two-hit framework, in which combined environmental stressors may result in greater cardiotoxicity [80].

#### 4.2.2. Pyroptosis

Pyroptosis is a form of inflammatory programmed cell death that has been recognized as a contributor to myocardial injury. Following ROS-mediated NF-κB activation, pyroptosis occurs after NLRP3 inflammasome assembly and caspase-1 activation. This process cleaves both gasdermin D (GSDMD) to form membrane pores, and simultaneously activates IL-1β and IL-18, amplifying the local inflammatory response [83]. Through this dual mechanism of membrane disruption and inflammatory recruitment, pyroptosis has been associated with cardiomyocyte loss, fibrosis, and hypertrophy [84].

Across rodents, avian, and human organoid models, PS-MPs and PS-NPs have been shown to consistently activate this pathway through ROS-mediated NF-κB and NLRP3 signaling, leading to caspase-1 activation and pyroptosis [47,53,80]. Another route separate from NLRP3 activation has been identified: impaired mitophagy. As described in Section 4.1.2, MNP-induced suppression of DKK3 impairs mitochondrial clearance, leading to the accumulation of damaged mitochondria and providing a ROS-independent signal for the activation of the NLRP3 inflammasome and pyroptosis [85]. The combination of oxidative stress and impaired mitophagy on an inflammatory death pathway is consistent with the interpretation that MNP cardiotoxicity may arise from an interconnected network of injury mechanisms.

#### 4.2.3. Ferroptosis

Ferroptosis is a form of regulated cell death characterized by iron-dependent lipid peroxidation and has been recognized as another mechanism of cardiomyocyte injury across several cardiomyopathy subtypes [86]. Its mechanism depends on the accumulation of lipid peroxides, which are produced when excess ferrous iron drives the Fenton reaction, generating hydroxyl radicals that attack polyunsaturated fatty acids (PUFAs) in cellular membranes [87]. Ferroptosis has also been implicated in doxorubicin-induced cardiomyopathy, in which mitochondrial iron accumulation, increased ROS production, and lipid peroxidation have been associated with cell death and impaired contractility [88].

MNP-induced ferroptosis occurs through two central molecular mechanisms. First, ROS generation promotes lipid radical formation and dysregulation of iron homeostasis, elevating the intracellular ferrous iron and increasing lipid peroxidation via the Fenton reaction [87]. Second, MNP exposure is associated with upregulation of ACSL4, an enzyme that incorporates PUFAs into the phospholipid membrane, which increases the amount of substrate for ferroptosis, whilst simultaneously reducing GPX4, a key antioxidant enzyme needed for detoxification of lipid peroxidation [89].

Upregulation of HIF-1 expression after MNP exposure has been shown to further promote ferroptosis, and pharmacological inhibition of HIF-1 was associated with reversal of both ferroptosis and fibrosis in the same model [87]. By increasing substrate availability and suppressing lipid peroxide defense, MNP-induced ferroptosis may bridge the oxidative stress described in Section 4.1 with the fibrotic remodeling addressed in Section 4.3.

### 4.3. Myocardial Fibrosis

Myocardial fibrosis is characterized by excessive deposition of extracellular matrix (ECM), including collagen I/III and fibronectin, leading to reduced myocardial compliance, impaired contractility, and increased arrhythmogenic potential [90]. Although cardiomyocyte apoptosis initially activates myofibroblasts for tissue repair, sustained activation leads to irreversible ECM accumulation and disruption of tissue architecture [91,92]. Clinically, fibrosis is identified in approximately 40% of DCM patients on late gadolinium enhancement (LGE) imaging, and in non-ischemic DCM, LGE-scarring was an independent predictor of mortality, establishing fibrosis as a prognostically meaningful marker [93,94].

Histological analyses have confirmed that MNPs accumulate within myocardial tissue and are associated with structural alterations, with apoptosis as the initiating stimulus for fibroblast activation [95]. Across multiple PET-MNP and PS-MNP models, ROS-driven mitochondrial dysfunction and apoptosis have been associated with significant increases in myocardial collagen deposition [47,58,78].

Two pro-fibrotic signaling pathways have been most consistently implicated. The Wnt/β-catenin pathway has been identified as a key mechanism in the pathogenesis of cardiac fibrosis [96]. After Wnt binds to its receptor, β-catenin is released from its degradation complex, translocates to the nucleus, and increases transcription of pro-fibrotic factors, such as collagen [97]. In rats exposed to PS-MPs for 90 days, activation of this pathway was associated with increased collagen I/III deposition, α-SMA, a marker of myofibroblast activation, and TGF-β [78]. Bridging cell death and fibrosis is DKK3, a negative regulator of Wnt signaling. MNP-mediated suppression of DKK3 not only activates the Wnt/β-catenin signaling pathway but also impairs mitophagy and activates the NLRP3-mediated pyroptosis, as described in Section 4.2.2 [85]. These overlapping mechanisms between fibrosis and pyroptosis further illustrate the complexity of the network of mechanisms underlying MNP-driven cardiotoxicity.

The second pro-fibrotic pathway is via TGF-β1/Smad2/3 signaling. Oral PS-NP administration in mice was associated with increased myocardial ROS, activation of the TGF-β1/Smad2/3 signaling pathway, and fibroblast proliferation and ECM accumulation [54]. This pathway was validated by prior work that replicated this pathway in angiotensin II-induced cardiac fibrosis [98].

MNPs exacerbate fibrosis in models of pre-existing myocardial injury. In cardiomyocytes co-exposed to PS-NPs and lipopolysaccharide, ROS generation and structural damage were greater than with either exposure alone, accompanied by increased TGF-β1 expression and fibrotic remodeling [54]. This augmented response is consistent with the two-hit model, whereby in cardiac tissue already compromised by genetic variants or ischemic injury, MNP exposure may lower the threshold for further fibrotic remodeling, although this remains to be demonstrated in human cohorts. Fibrosis driven by these pathways not only represents a terminal endpoint of cellular injury but is also linked to functional impairment, as described in the following section.

### 4.4. Cardiac Injury and Functional Consequences

The cellular injury cascade described in Section 4.1, Section 4.2 and Section 4.3 has also been associated with functional impairment as assessed by imaging. Emerging evidence suggests that MNP-induced cardiac remodeling is dose-, duration-, and route-dependent and begins with an initial stress response that progresses to a dilated systolic dysfunction phenotype consistent with DCM in animal models.

#### 4.4.1. Initial Stress Response

Early on, MNP exposure in murine models results in a transient stress response with preserved or modestly enhanced systolic function. Mice exposed to inhaled PS-NPs for one and two weeks were found to have increased EF and SV, accompanied by an increased LV myocardial mass and ECG evidence of QRS broadening [99]. These findings of a hyperdynamic profile are consistent with acute sympathetic stress activation in response to systemic injury, similar to the state of excess catecholamine or early sepsis, where systolic function may overcompensate prior to myocardial depression. When PS-MPs were delivered at low concentrations via intratracheal instillation for four weeks, there was a significant reduction in ventricular cavity area and increased interventricular septal thickness [56]. Upregulation of MYH7B, MYL2, MYL4, and CX43 was observed, and these changes closely mirrored those seen with the positive isoproterenol controls, suggesting stress response activation rather than pathological hypertrophy.

#### 4.4.2. Transition to Dilated Cardiomyopathy Phenotype

Prolonged or higher-dose exposure of MNPs may tip the balance from adaptation to cellular destruction. The proposed driver of this transition is the progressive loss of cardiomyocytes through apoptosis, pyroptosis, and ferroptosis, which may lead to eccentric LV remodeling and increased wall stress on the remaining contractile units in the myocardium. This progression is both temporal and dose-dependent. In a study using multiple doses and time points, EF and FS were preserved at one week but declined significantly in a dose-dependent manner by week four, with partial recovery at low and medium doses by week twelve [52]. Cardiac fibrosis was absent at 4 weeks but developed significantly by week 12, suggesting that fibrosis may lag behind the initial functional impairment and potentially serve as a transition point before further deterioration. LV cavity dimensions increased with dose and duration, and transcriptomic analysis at the end of the study was enriched for dilated cardiomyopathy and heart failure gene sets, as well as mitochondrial dysfunction pathways [52].

Three independent mechanisms converge on this fibrosis endpoint. The first pathway involves p53-mediated apoptosis and TGF-β1/Smad3-driven myofibroblast differentiation, with a dose-dependent hierarchy: apoptosis at medium dose and combined apoptosis and fibrosis activation at high doses [100]. Another proposed mechanism is the activation of the HIF/ROS/GPX4 ferroptosis axis, where pharmacological inhibition of ferroptosis was associated with reduced fibrosis-related gene expression in human AC16 cardiomyocytes, supporting a potential causal role for ferroptosis in fibrosis [87]. The third pathway involves DKK3 suppression and activation of the NLRP3-mediated pyroptosis [85]. Despite their different molecular origins, all three pathways have been associated with interstitial collagen deposition and eccentric remodeling.

The DCM phenotype is reproduced across multiple models. When PS-NPs were administered for 42 days, this was associated with dilated ventricular chambers, thinned posterior walls, and a dose-dependent reduction in EF in mice [46]. PE-NP exposure over four weeks produced dose-dependent reductions in EF from 70.5% to 58.1% and FS from 37.5% to 24.4% in the high-dose group [85]. Similarly, inhaled PS-MP exposure over six weeks produced comparable systolic and diastolic impairment [87]. The reproducibility of this phenotype across polymer types and exposure routes is consistent with a degree of polymer-agnostic cardiotoxicity, but physicochemical differences between polymers may also contribute to this observed effect, as discussed in Section 4.1.1. The key murine studies demonstrating functional cardiac impairment following MNP exposure are summarized in Table 1.

#### 4.4.3. Two-Hit Framework and Clinical Relevance

MNP exposure has been shown in preclinical models to exacerbate pre-existing myocardial injury, with a greater functional impairment observed compared to either exposure alone. In mice with isoproterenol-induced myocardial stress, combined PS-NP exposure resulted in greater reductions in systolic function, greater apoptosis, and fibrosis [100]. These findings raise the hypothesis that individuals with established DCM, underlying genetic susceptibility, or co-exposure to other environmental toxins may be more vulnerable to MNP-induced cardiac deterioration.

In summary, we describe a mechanistic pathway derived from preclinical studies, in which MNP exposure initially produces an acute stress response with preserved systolic function, but, following prolonged or higher-dose exposure, may promote progressive cardiomyocyte loss, myocardial fibrosis, and eccentric LV remodeling consistent with a DCM phenotype. The overall strength of these pathways varies in terms of consistency and translational evidence, as summarized in Supplementary Table S1.

## 5. Discussion

### 5.1. Summary of Key Findings

In summary, based on preclinical evidence, we propose a sequential model starting with cellular injury, which may lead to dysregulation of tissue structure and fibrosis, as supported by functional impairment observed on imaging. The initial trigger of this cascade is oxidative stress, which also perpetuates the other cellular mechanisms. Across multiple polymer types, MNP has been associated with elevated ROS, reduced antioxidant defense, and the activation of the NF-κB and p38/MAPK signaling cascades, which are recognized mediators of heart failure progression. In addition, oxidative stress disrupts normal mitochondrial and ER homeostasis by dysregulating calcium via SERCA inhibition. This prolongs cytosolic calcium exposure, which is subsequently transferred to the mitochondria, potentially leading to reduced energy metabolism and increased ROS generation, reinitiating the cycle. ER stress compounds this damage by activating pro-apoptotic signals via CHOP and XBP-1 and impairing autophagic flux. From these interconnected pathways emerge various forms of cardiomyocyte death, including apoptosis via the mitochondrial pathway, pyroptosis via the NLRP3 inflammasome, and ferroptosis via lipid peroxidation and GPX4 suppression. At the tissue level, prolonged cell death has been associated with myocardial fibrosis via the Wnt/β-catenin, TGF-β1/Smad2/3, and DKK3 pathways in preclinical models. The shared role of DKK3 in pyroptosis and fibrosis further reinforces the interconnected nature of these pathways.

We explored the functional consequences of this injury model. The extent of injury depended on dose and duration, and the pathogenesis was closely associated with other toxic cardiomyopathies. Lower doses and shorter exposure times were associated with a transient stress response with a preserved and mild increase in systolic function, which may reflect a sympathetic response. Following prolonged exposure and higher doses, greater cardiomyocyte loss and increased fibrosis were observed in preclinical models. On echocardiographic assessment in murine models, eccentric remodeling with chamber dilation, posterior wall thinning, and dose-dependent reductions in ejection fraction and fractional shortening were observed, consistent with a dilated cardiomyopathy phenotype. In addition, transcriptomic analysis from a twelve-week murine exposure model was enriched for dilated cardiomyopathy and heart failure gene sets, providing molecular evidence for these functional findings. Consistent with the two-hit framework, greater functional impairment was observed in models of pre-existing stress. Specifically, the combination of MNP exposure and hemodynamic stress resulted in a greater reduction in systolic function than with either exposure alone.

### 5.2. Contextualizing Within the Exposome-Driven Cardiotoxicity

Although epidemiological studies directly investigating MNP exposure and heart failure outcomes remain limited, the translation pathway from preclinical mechanistic evidence to population-level risk has been well established for air pollution and heavy metals, providing a useful precedent for the trajectory the MNP field may follow.

#### 5.2.1. Air Pollution

Air pollution is a major environmental health threat associated with increased HF incidence, progression, and adverse outcomes. Particulate matter, specifically PM_2.5_ (particles < 2.5 µm), is the most extensively studied. After inhalation, PM_2.5_ reaches the alveolar space, enters systemic circulation, and induces oxidative stress, endothelial injury, and systemic inflammation. This cascade is mechanistically similar to that described for MNPs in Section 4.1, with calcium homeostasis among the most closely shared injury mechanisms. Chronic PM_2.5_ exposure in a murine model resulted in downregulation of SERCA2a expression and reduced cardiac contractile function on echocardiography [101]. This shared mechanism between MNPs and PM_2.5_ suggests that SERCA-mediated calcium dysregulation may represent a shared pathway of environmentally mediated cardiac injury.

The association between air pollution and adverse outcomes in DCM has also been shown in clinical studies. Long-term exposure to air pollution in patients with established DCM was independently associated with greater LV mass and lower ejection fraction [102]. In addition, a cardiac MRI study found that long-term exposure to ambient fine particle air pollution was associated with greater diffuse myocardial fibrosis in DCM patients than in healthy controls [103]. At a population level, data from the UK Biobank and the Ontario cohort found that long-term exposure to combined airborne pollutants was associated with an elevated risk of heart failure [104,105]. In addition, a meta-analysis of 35 studies found that each 10 μg/m^3^ increase in PM_2.5_ was associated with a 2.12% increase in heart failure hospitalizations or deaths [106]. EPA registry data similarly found a 13% increase in all-cause mortality per 1 μg/m^3^ in both systolic and diastolic heart failure populations. This breadth of evidence, spanning cellular mechanisms, animal models, population cohorts, and meta-analyses, illustrates the trajectory toward which MNP research is moving but has yet to achieve.

#### 5.2.2. Heavy Metal Exposure

Heavy metal exposure represents another established environmental cardiotoxicant that shares mechanistic pathways with MNP-induced injury. Cadmium and lead are both associated with heart failure and share overlapping mechanistic injury patterns with MNPs, including oxidative stress, mitochondrial dysfunction, and calcium dysregulation.

Cadmium, which is released from industrial emissions and contaminated water sources, disrupts the intracellular balance of calcium and zinc [107]. It also induces oxidative stress, chronic inflammation, mitochondrial dysfunction, and fibrosis [108]. In a murine model, cadmium downregulated SERCA2a protein expression and reduced phosphorylation of phospholamban, which was associated with impaired sarcoplasmic reticulum calcium reuptake and reductions in ejection fraction and fractional shortening [109]. This SERCA2a-mediated contractile dysfunction is a mechanism shared by both MNPs and PM_2.5_, making cadmium the third independent exposure to be associated with sarcoplasmic reticulum calcium dysregulation and cardiomyocyte injury. Lead exposure has additionally been associated with reduced ejection fraction, increased left ventricular volume, impaired mitochondrial function, and inhibition of voltage-gated calcium channels [110,111].

Epidemiological evidence linking cadmium to heart failure is consistent across multiple cohorts. Among over 10,000 participants across the MESA, Strong Heart Study, and Hortega cohorts, increased urinary cadmium was independently associated with increased heart failure incidence [10]. These findings are further supported by data from 12,049 participants in the NHANES study, which found that a 50% increase in blood cadmium level was associated with a 48% increased risk of heart failure [108]. The Malmö Diet and Cancer study found that the highest cadmium quartile was associated with approximately double the risk of heart failure compared to the lowest quartile [107]. Regarding DCM, an observational study in the Democratic Republic of Congo found that exposure to heavy metals was associated with an increased likelihood of DCM [112]. These findings suggest that environmental heavy metal exposure may carry consequences specific to DCM pathogenesis, though the observational design of the available studies limits causal inference.

### 5.3. Mechanistic Similarities with Established Cardiovascular Toxins

Alcohol, anthracyclines, and viral myocarditis are well-established triggers of DCM, each resulting in eccentric LV remodeling and systolic dysfunction through mechanisms with substantial overlap with the MNP injury cascade that has been described in this review. By examining these toxins, we contextualize MNP-induced cardiotoxicity within established frameworks and propose that the mechanisms linking MNP to cardiomyopathy are not novel in themselves but are novel in their environmental source. Consistent with the two-hit hypothesis, marked variation in the extent of damage at equivalent exposure has been observed across studies, suggesting that factors such as genetic susceptibility modulate phenotypic expression. This framework is supported by a longitudinal study of familial DCM, which found that DCM-promoting factors determined phenotypic penetrance in genotype-positive individuals and were responsible for LVEF fluctuations in the majority of cases, especially in patients with TTNtv, who showed uniform susceptibility to these triggers [5].

#### 5.3.1. Alcohol Induced Cardiomyopathy

Alcohol induced cardiomyopathy (AC) is an acquired form of DCM that is caused by prolonged and heavy alcohol consumption in the absence of other causes [113]. It represents one of the main causes of non-ischemic dilated cardiomyopathy and is accountable for around one-third of cases [114]. Its cardiotoxic effects are mediated through ethanol and acetaldehyde toxicity. Ethanol activates NOX2 and Ca/calmodulin-dependent kinase II (CaMKII), inducing SR calcium leak with downstream arrhythmogenic and negative inotropic effects [114]. Excess CaMKII in murine models has been associated with increased cell death, fibrosis, and ventricular dilation [115]. This NOX2/CaMKII axis may represent another pathway of damage following the TLR4/NOX2 activation as described in Section 4.1.1. Acetaldehyde compounds this injury by generating mitochondrial ROS, activating p38/MAPK, ERK, and JNK signaling, and impairing SR calcium release and the excitation-contraction link [116]. This combined effect has been associated with mitochondrial ETC disruption, Bax- and caspase-driven apoptosis, fibrosis, and adverse remodeling [117]. Patients with TTNtv experienced an 8.7% greater absolute reduction in LVEF than those without, despite equivalent exposure [118]. The recurrence of TTNtv as a modifier across mechanistically distinct cardiotoxins raises the possibility that a similar genetic vulnerability may exist in the context of MNP and other environmental exposures, though this remains to be investigated.

#### 5.3.2. Anthracycline Cardiomyopathy

Anthracyclines are a group of chemotherapy agents that are the cornerstone of treatment of many malignancies, but following sustained use, may lead to cardiomyopathy [119]. They provide molecularly specific parallels with MNP-induced cardiotoxicity with similarities in both oxidative stress pathways and cell death mechanisms. Anthracyclines induce damage through two pathways. The first mechanism is through interference with DNA topoisomerase 2, which increases DNA strand breaks and activates p53-mediated cardiomyocyte cell death via the intrinsic mitochondrial pathway [119,120]. The second, which is more relevant to this review, involves binding to cardiolipin on the inner mitochondrial membrane, disrupting the ETC, and reducing antioxidant capacity [119,121]. Anthracyclines have also been shown to reduce the thiol groups of SERCA2A, impairing calcium reuptake and resulting in mitochondrial calcium overload, mPTP opening, reduced membrane potential, and reduced ATP synthesis [121]. Iron accumulation has also been implicated in anthracycline-induced cardiac injury, potentially driving the Fenton reaction, suppressing GPX4 through Nrf2 inhibition, and triggering ferroptosis via the same ACSL4/GPX4 axis described in Section 4.2.3. The clinical consequences are well established and include progressive LV dysfunction with chamber dilation, wall thinning, decreased EF, and decreased fractional shortening [119].

Genome-wide association studies have also identified genetic variants that increase susceptibility to chemotherapy-induced cardiomyopathy, consistent with the two-hit hypothesis [4]. In three independent cohorts of patients with chemotherapy-induced cardiomyopathy, 7.5% were found to have TTNtv compared with less than 1% in the controls [122]. In addition, TTNtv carriers experienced greater heart failure hospitalization, atrial fibrillation, and reduced LVEF recovery [122]. TTNtv, as a modifier for alcohol and anthracycline-induced cardiomyopathy, is consistent with this particular variant, lowering the threshold for DCM phenotype expression following cardiotoxic insult, and raises the question of whether similar susceptibility may be observed for MNP-induced myocardial injury.

#### 5.3.3. Viral Myocarditis and Inflammatory Cardiomyopathy

Viral myocarditis is an important framework for this review in which an acute environmental trigger can result in an irreversible DCM phenotype in a susceptible host. It is most commonly caused by cardiotropic viruses, including Coxsackievirus B and Parvovirus B19, which inflict both direct cytotoxic and immune-mediated mechanisms [123]. In a subset of patients, acute inflammation does not resolve and progresses to chronic inflammatory DCM, characterized by cardiomyocyte loss, fibrotic remodeling, and LV systolic dysfunction [124]. Enteroviral proteases 2A and 3C cleave dystrophin and other cytoskeletal proteins, impairing contractile force and promoting apoptosis [125]. A transgenic mouse with low-level expression of the Coxsackie B virus genome, without active replication, demonstrated molecular, histological, and functional impairment consistent with a DCM phenotype [126]. This suggests that chronic cardiomyocyte injury may promote a DCM phenotype in the context of persistent viral genome expression.

Another mechanism that is shared with MNP-induced injury is TLR4 activation. Activation of TLR4 on dendritic cells is required for the induction of acute myocarditis and progression to heart failure [81]. TLR4-deficient mice showed significantly reduced myocarditis severity and viral replication following Coxsackievirus B3 infection [127]. In human DCM, myocardial TLR4 expression has been associated with enterovirus replication and LV dysfunction [128]. This is consistent with its role as a mediator of NOX2-driven ROS and NF-κB-mediated cardiomyocyte injury, as described in Section 4.1.1. TLR4 activation in both viral and MNP-induced injury has been associated with TNF release and oxidative stress, which may contribute to adverse cardiac remodeling.

This transition from myocarditis to DCM is consistently influenced by genetic predisposition. Genetically distinct mice inoculated with the same virus showed varying responses depending on polymorphisms in immune response pathways. In human studies, genetic variants of cytoskeletal proteins, including dystrophin and desmoplakin, have been linked with greater susceptibility to virus-induced DCM. This could be explained by the way viral proteases cleave cytoskeletal proteins, which, when genetically disrupted, may predispose to cardiomyopathy [129]. It remains uncertain, however, whether viral infection directly causes DCM or merely accelerates the transition in individuals with greater genetic susceptibility.

**Figure 2:**
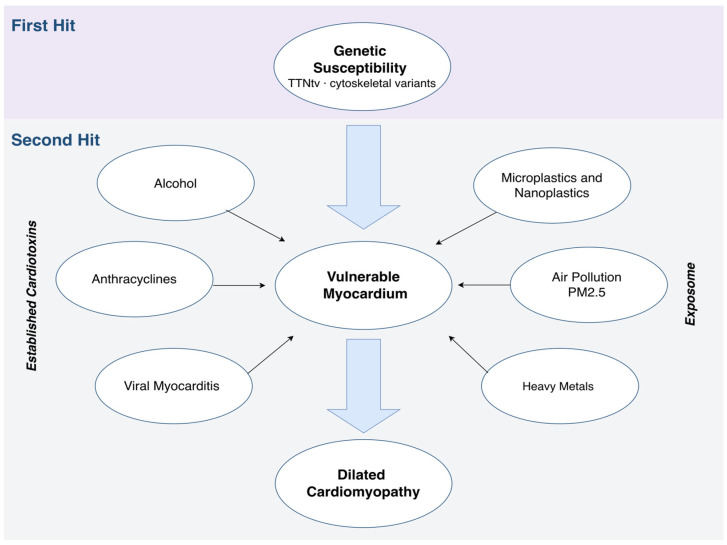
The two-hit model of dilated cardiomyopathy: genetic susceptibility and environmental and cardiotoxic second hits.

In summary, across all three cardiotoxins, a consistent pattern of injury emerges. DCM preferentially occurs in those who are genetically susceptible, particularly TTNtv carriers. This recurrence across mechanistically distinct cardiotoxins provides evidence that TTNtv may lower the threshold for DCM phenotype expression. Individuals carrying this genotype may therefore represent a high-risk population who could be disproportionately affected by chronic MNP exposure. The shared mechanisms across exposures, within the exposome framework, provide justification for considering MNP exposure as a potential contributor in the evaluation of unexplained DCM. The mechanistic parallels and clinical evidence across these exposures are summarized in Table 2. The proposed two-hit model integrating genetic susceptibility with established cardiotoxins and novel exposomic factors is illustrated in Figure 2.

Dilated cardiomyopathy (DCM) arises from complex gene-environment interactions. We present a two-hit model in which TTNtv and other cytoskeletal genetic variants act as the first hit, lowering the phenotypic threshold for decompensation. In genetically susceptible individuals, a second hit is required to cross this threshold for the transition to overt DCM. Second hits can be categorized into established cardiotoxins, including alcohol, anthracyclines, and viral myocarditis, and exposomic factors, including air pollution (PM_2.5_), heavy metals, and microplastics and nanoplastics (MNPs). MNPs are novel and previously underrecognized components of the exposome and share similar cellular injury pathways as established cardiotoxins. All second hits lead to a vulnerable myocardium characterized by cardiomyocyte loss, myocardial fibrosis and eccentric left ventricular remodeling, mirroring the DCM phenotype.

### 5.4. Limitations and Translational Challenges

The preclinical evidence presented in this narrative review provides mechanistic insights and a biologically plausible case for MNP-induced DCM. However, definitive evidence for a causal relationship requires critical appraisal before these findings inform clinical practice.

A central limitation is the absence of prospective human data linking MNP exposure to DCM. Most evidence is derived from in vitro and animal models. Even the more recent cardiac organoid and hiPSC-CM data do not replicate the complexity of the human cardiovascular environment, which includes immune regulation, hemodynamic forces, and comorbidities. The available human observational data, which have primarily been used in the surgical population, cannot establish causality, as they represent a pre-selected high-risk population in which it cannot be determined whether MNP accumulation initiates or is a consequence of pre-existing pathological changes. Furthermore, it is important to distinguish the available evidence of general cardiovascular toxicity, including MNP detection in atherosclerotic plaques and associations with major adverse cardiovascular events, from DCM remodeling, for which direct human evidence remains absent. Establishing causality is further complicated by confounding. MNP exposure often co-exists with socioeconomic, dietary, occupational, and environmental factors, which themselves individually contribute to cardiovascular disease. Prospective longitudinal cohort studies enrolling healthy individuals with baseline MNP exposure assessment, longitudinal tracking of cardiovascular outcomes, and rigorous multivariable adjustment for co-exposures and traditional risk factors will be required before causation can be established.

There are also methodological challenges that limit the interpretability of the mechanisms. The majority of studies use polystyrene nanoparticles, which do not reflect the complex polymer composition of real-world human MNP exposure and also do not account for weathering and adsorbed co-contaminants that impact cytotoxicity. MNPs may additionally act as vectors for plasticizers, heavy metals, and polycyclic aromatic hydrocarbons, which, when combined, may further increase cardiotoxicity through overlapping mechanisms and are not accounted for by single-polymer experimental models. Physicochemical heterogeneity across polymer types is therefore an underappreciated source of complexity. MNPs vary in polymer type, size, shape, surface charge and weathering which all modulate cytotoxicity independently. Treating MNPs as a uniform exposure risk obscures biologically meaningful differences between particle types. Many studies use high-dose, acute exposure protocols that do not reflect the chronic, low-level accumulation more in line with real-world environmental exposure. The doses used in most animal studies exceed the estimated human blood concentrations of approximately 1.6 μg/mL and it remains uncertain whether the phenotypic consequences observed at experimental doses are reproduceable at environmentally relevant concentrations. As discussed in Section 4.4, dose and duration are unlikely to reflect the consequences of decades of low-level accumulation. Another major factor currently reducing the quality of existing evidence is the lack of standardization in MNP detection and characterization. Current analytical techniques, including µFTIR, Raman spectroscopy, and Py-GC/MS, vary in detection thresholds, size resolution, and polymer specificity, which results in interstudy variability.

### 5.5. Future Directions

Future research most importantly requires chronic, low-dose exposure models that better reflect real-world human MNP exposure. Long-term animal studies using environmentally relevant particle concentrations, polymer mixtures, and exposure durations should be designed to further elucidate the dose and time-dependent phenotypic trajectory described in Section 4.4. Human-relevant experimental systems, including cardiac organoids and iPSC-derived cardiomyocytes, should be prioritized to bridge the current gap between animal data and human physiology. Further work is also needed to investigate the role of particle size, polymer type, surface charge, and adsorbed co-contaminants in determining uptake, tissue penetration, and cardiotoxicity, given that physicochemical heterogeneity across MNP types may modulate the biological effects.

Development of internationally accepted protocols for MNP isolation, quantification, and classification across diverse biological matrices would reduce inter-study variability and provide consistent metrics to facilitate large-scale cohort studies. Alternative exposure biomarkers that measure specific metabolites or additives in blood or urine may provide a practical, scalable tool for epidemiological studies.

Genomic, epigenomic, and metabolomic profiling should be incorporated into future experimental and epidemiological studies to identify early biomarkers of MNP-induced myocardial injury and to characterize the gene-environment interactions that determine susceptibility. Given that TTNtv carriers are more susceptible to cardiotoxins, a prospective study design comparing high versus low MNP exposure in genotype-positive, phenotype-negative individuals, assessed using serial echocardiography, cardiac MRI, biomarkers, and validated MNP exposure assays, would represent a logical next step to address this translational gap.

A prospective observational multicenter study is currently addressing several of these translational priorities. Myocardial tissue and peripheral blood collected during cardiac surgery will be analyzed by Py-GC/MS and electron microscopy, and cardiac function will be assessed by comprehensive echocardiography, including three-dimensional assessment and speckle-tracking analysis, to identify a potential imaging signature of MNP exposure [95]. The results from this trial may represent early steps towards prospective human data linking MNP exposure to in vivo functional impairment. Speckle-tracking-derived global longitudinal strain may detect subclinical systolic dysfunction before LVEF decline, and its inclusion could help identify an early functional signature of MNP-induced myocardial injury that precedes overt evidence of DCM on echocardiography.

From a public health standpoint, MNPs warrant consideration for integration into the exposome framework for cardiovascular risk assessment alongside established contributors, including PM_2.5_ and heavy metals. Regulatory action should be taken to reduce environmental plastic contamination through source reduction, safer polymer alternatives, and improved waste management, combined with guidance on personal exposure reduction. These steps would represent a rational precautionary response to the current mechanistic evidence, acknowledging that definitive proof of causation in human populations is not yet available. Within the two-hit model proposed in this review, individuals carrying TTNtv and other DCM-causing variants may constitute a subgroup warranting additional clinical consideration as environmental MNP exposure continues to rise.

## 6. Conclusions

This review synthesizes the preclinical mechanistic evidence suggesting that MNPs may contribute to myocardial injury as a plausible environmental second hit in the pathogenesis of DCM. Across preclinical models, oxidative stress emerges as the initial trigger that impairs mitochondrial function and induces ER stress via calcium dysregulation, potentially resulting in cardiomyocyte death, fibrosis, and eccentric LV dysfunction, consistent with a DCM phenotype. These mechanisms are consistent with those of established environmental cardiotoxins and recognized triggers of toxic cardiomyopathy. TTNtv is a recurring modifier across cardiotoxins that is sensitive to environmental stressors and may represent a specific group in which a window of phenotypic plasticity exists, in which myocardial function may be reversibly altered by modifiable exposures, and preventive measures could help slow disease onset. However, translational work still remains before applying these findings to clinical practice. Nevertheless, the biological plausibility of the mechanistic evidence, combined with the trajectory of global plastic production, provides justification for incorporating MNPs within the exposome framework of cardiovascular disease and for prioritizing prospective human investigation into their potential role in the pathogenesis of DCM.

## Figures and Tables

**Table 1:** Models demonstrating functional cardiac impairment following MNP exposure.

Study	Model	Exposure Route	Polymer Type	Dose	Duration	Key Functional Findings
[99]	ICR mouse, 5 wk, male	Inhalation	PS-NPs, 100 nm	28.4 mg/m^3^ (~1 mg/day)	1–2 weeks	↑ EF (+13%), ↑ SV (+43%), ↑ LV myocardial mass, ↑ LVIDd/LVIDs; QRS broadening on ECG → hyperdynamic/hypertrophic phenotype (acute stress response)
[56]	BALB/c mouse, 8 wk, male; hPSC cardiac organoids	Inhalation	PS-MPs, 1 µm	25, 50 µg × 2/week (mice); 0.025–2.5 µg/mL (organoids)	4 weeks (mice); 72 h (organoids)	↓ Ventricular cavity area, ↑ IVST → concentric hypertrophy pattern; ↑ MYH7B, ANP, BNP, COL1A1 in organoids
[52]	C57BL/6 mouse, 6 wk, male	Inhalation	PS-NPs, 40 nm	16, 40, 100 µg/day (low, medium, high)	1, 4, 12 weeks	EF/FS preserved at 1 week; dose-dependent ↓ EF/FS by 4 weeks; partial recovery at low/ medium dose by 12 weeks; ↑ LVIDd/LVIDs with dose and duration; transcriptomic enrichment for DCM and HF gene sets at 12 weeks
[100]	C57BL/6 mouse, 6 wk, male	Oral	PS-NPs, ~84 nm	0.1, 0.5, 2.5 mg/day (low, medium, high)	6 weeks	High-dose: ↓ EF/FS; no hypertrophy; isoproterenol co-exposure produced greater ↓ systolic function than either alone
[87]	C57BL/6J mouse, 6 wk, male; AC16 human cardiomyocytes	Inhalation	PS-MPs, 5 µm	100 µg/dose every 5 days (×12 doses)	~60 days	↓ EF/FS, ↓ LVAW, ↓ LVPW; systolic and diastolic impairment; pharmacological HIF-1 inhibition reversed ferroptosis and fibrosis
[46]	C57BL/6J mouse, 6–8 wk, male; H9C2 cells	Oral	PS-NPs, ~90 nm	30, 60, 100 mg/L	42 days	↑ LVIDd/LVIDs, ↓ wall thickness, dose-dependent ↓ EF/FS, ↓ HR, ↓ BP → explicit DCM phenotype; dilated ventricular chambers with thinned posterior walls
[85]	C57BL/6 mouse, 7–8 wk, male; HL-1 cardiomyocytes	Oral	PE-NPs (DS100), 100 nm	30, 60, 100 mg/kg	4 weeks	Dose-dependent ↓ LVEF (70.5% → 58.1%) and ↓ LVFS (37.5% → 24.4%) at high dose

↑ denotes increase. ↓ denotes decrease.

**Table 2:** Mechanisms and myocardial injury across environmental exposures and established cardiotoxins.

Mechanism	MNPs	PM_2.5_	Heavy Metals	Alcohol	Anthracyclines	Viral Myocarditis
Oxidative Stress	TLR4/NOX2 → ↑ ROS ↓ SOD · GSH-Px · CAT NF-κB · p38/MAPK activation	Systemic ROS · endothelial injury Shared NF-κB-mediated inflammation	Cd: direct ROS · ↓ antioxidants Pb: ROS mitochondrial impairment	Ethanol: NOX2/CaMKII Acetaldehyde: mitochondrial ROS p38/MAPK · ERK · JNK activation	Cardiolipin binding → ETC disruption ↓ Antioxidant capacity Shared NOX2 axis with MNPs	TLR4 → NF-κB · TNF-α Immune-mediated ROS
Mitochondrial Dysfunction	↓ MMP · ↓ ATP synthesis Cytochrome C release ↓ DKK3 → impaired mitophagy cGAS-STING	Implied via shared ROS pathways Not directly described	Cd: mitochondrial dysfunction ↓ Intracellular Ca^2+^/zinc balance Pb: impaired mitochondrial function	Acetaldehyde → ETC disruption ↓ ATP · Bax/caspase apoptosis	Cardiolipin binding → ↓ MMP mPTP opening · ↓ ATP Mitochondrial Ca^2+^ overload	Less directly implicated Cytoskeletal cleavage → energetic failure
Calcium Dysregulation	SERCA inhibition → ↑ cytosolic Ca^2+^ SR Ca^2+^ → mitochondrial overload Possible membrane adsorption	SERCA2a ↓ → ↓ SR Ca^2+^ reuptake	Cd: SERCA2a ↓ · phospholamban ↓ Pb: voltage-gated Ca^2+^ channel ↓ SERCA-mediated pathway	CaMKII → SR Ca^2+^ leak Acetaldehyde → ↓ EC coupling Arrhythmogenic · negative inotropy	SERCA2A thiol oxidation → ↓ reuptake Mitochondrial Ca^2+^ overload → mPTP	Not directly described Cytoskeletal impairment → ↓ contractile force
ER Stress	UPR activation CHOP · XBP-1 Pro-apoptotic gene expression Impaired autophagic flux	—	—	Partial via acetaldehyde toxicity Not described as a distinct pathway	SERCA2A disruption Downstream Ca^2+^ effects on ER	—
Cardiomyocyte Death	Apoptosis: Bax/BCL2 ↑ · caspase-9 Pyroptosis: NLRP3 · caspase-1 · GSDMD Ferroptosis: ↓ GPX4 · ↑ ACSL4	Apoptosis via shared oxidative and inflammatory pathways	Cd: apoptosis via oxidative stress Pb: apoptosis	Apoptosis: Bax · caspase-9 via the CaMKII/NOX2 axis	Apoptosis: p53-mediated intrinsic pathway Ferroptosis: ACSL4/GPX4 axis	Apoptosis: direct cytotoxic + immune-mediated Proteases cleave dystrophin
Myocardial Fibrosis	Wnt/β-catenin DKK3 suppression TGF-β1/Smad2/3 signaling HIF-1/ROS/GPX4 ferroptosis axis	Diffuse fibrosis on cardiac MRI in DCM patients [103]	Cd: fibrosis via oxidative stress and mitochondrial injury	Acetaldehyde → myofibroblast activation Eccentric remodeling collagen ↑	p53-apoptosis → myofibroblast activation Ferroptosis → fibrosis-related gene ↑	Chronic inflammatory DCM subset: cardiomyocyte loss → fibrotic remodeling
DCM Phenotype and Human Evidence	↓ EF · ↑ LVIDd eccentric remodeling Fibrosis by week 12 in the murine model [52] Transcriptomics enriched for DCM gene sets No prospective human data	↑ LV mass · ↓ EF in DCM patients Diffuse fibrosis on cardiac MRI [103] +2.12% HF events per 10 μg/m^3^ PM2.5 [106]	Cd: urinary level → ↑ HF across MESA · Strong Heart · Hortega · [129] NHANES 50% ↑ blood Cd → 48% ↑ HF risk [108] DCM association: DRC observational study [112]	~1/3 of non-ischemic DCM cases [114] TTNtv: 8.7% greater absolute ↓ LVEF vs. non-carriers at equivalent exposure [118]	↓ EF · chamber dilation · wall thinning TTNtv in 7.5% vs. <1% controls (three independent cohorts) [122] ↑ HF hospitalization · AF · ↓ LVEF recovery in TTNtv carriers [122]	Progression to inflammatory DCM in a subset of acute myocarditis Cytoskeletal variants (dystrophin, desmoplakin) ↑ susceptibility [129]

↑ denotes increase, ↓ denotes decrease.

## Data Availability

No new data were created for the purpose of this study.

## Supplementary Materials

The following supporting information can be downloaded at: https://www.mdpi.com/article/10.3390/life16060916/s1. Table S1: Preclinical Evidence Grade for MNP induced Myocardial Injury Pathways.

## Abbreviations

The following abbreviations are used in this manuscript:

AC	Alcohol-induced cardiomyopathy
ACSL4	Acyl-CoA synthetase long-chain family member 4
AMPK ANP	AMP-activated protein kinase Atrial natriuretic peptide
ATP	Adenosine triphosphate
Bax	BCL2-associated X protein
BCL2	B-cell lymphoma 2
BNP	Brain natriuretic peptide
CaMKII	Ca^2+^/calmodulin-dependent protein kinase II
CAT	Catalase
cGAS-STING	Cyclic GMP-AMP synthase stimulator of interferon genes
CHOP	C/EBP homologous protein
CK-MB	Creatine kinase-MB
cTnI	Cardiac troponin I
cTnT	Cardiac troponin T
CX43	Connexin 43
DAMP	Damage-associated molecular pattern
DCM	Dilated cardiomyopathy
DKK3	Dickkopf-related protein 3
ECG	Electrocardiogram
ECM	Extracellular matrix
EF	Ejection fraction
EPA	Environmental Protection Agency
ER	Endoplasmic reticulum
ERK	Extracellular signal-regulated kinase
ETC	Electron transport chain
FS	Fractional shortening
FTIR	Fourier transform infrared spectroscopy
GPX4	Glutathione peroxidase 4
GSDMD	Gasdermin D
GSH-Px	Glutathione peroxidase activity
HF	Heart failure
HIF-1	Hypoxia-inducible factor 1
HIPK2	Homeodomain-interacting protein kinase 2
hiPSC-CM	Human-induced pluripotent stem-cell-derived cardiomyocyte
IL-1β	Interleukin-1 beta
IL-18	Interleukin-18
JNK	c-Jun N-terminal kinase
LGE	Late gadolinium enhancement
LV	Left ventricle/left ventricular
LVIDd	Left ventricular internal diameter at diastole
LVIDs	Left ventricular internal diameter at systole
LVEF	Left ventricular ejection fraction
MAPK	Mitogen-activated protein kinase
MDA	Malondialdehyde
MESA	Multi-Ethnic Study of Atherosclerosis
MMP	Mitochondrial membrane potential
MNPs	Microplastics and nanoplastics
MPs	Microplastics
mPTP	Mitochondrial permeability transition pore
MRI	Magnetic resonance imaging
mtDNA	Mitochondrial DNA
MYH7B	Myosin heavy chain 7B
MYL2	Myosin light chain 2
MYL4	Myosin light chain 4
NADPH	Nicotinamide adenine dinucleotide phosphate
NF-κB	Nuclear factor kappa B
NHANES	National Health and Nutrition Examination Survey
NLRP3	NOD-like receptor protein 3
NOX	NADPH oxidase
NOX2	NADPH oxidase 2
Nrf2	Nuclear factor erythroid 2-related factor 2
NPs	Nanoplastics
p53	Tumor protein p53
PE	Polyethylene
PET	Polyethylene terephthalate
PGC-1α	Peroxisome proliferator-activated receptor gamma coactivator 1-alpha
PLB	Phospholamban
PM2.5	Particulate matter smaller than 2.5 micrometers
PMMA	Polymethylmethacrylate
PP	Polypropylene
PS	Polystyrene
PS-MPs	Polystyrene microplastics
PS-NPs	Polystyrene nanoplastics
PUFA PVC	Polyunsaturated fatty acid Polyvinylchloride
Py-GC/MS	Pyrolysis gas chromatography–mass spectrometry
ROS	Reactive oxygen species
SERCA	Sarco-endoplasmic reticulum calcium ATPase
SERCA2a	Sarco-endoplasmic reticulum calcium ATPase 2a
SOD	Superoxide dismutase
SR	Sarcoplasmic reticulum
SV	Stroke volume
TGF-β1	Transforming growth factor beta 1
TLR4	Toll-like receptor 4
TNF-α	Tumor necrosis factor alpha
Top2β	Topoisomerase IIβ
TTN	Titin
TTNtv	Titin truncating variants
UPR	Unfolded protein response
Wnt	Wingless-related integration site
XBP-1	X-box binding protein 1
