# Supplementary material for: Micro- and Nanoplastics as Potential Drivers of Dilated Cardiomyopathy
Source: Life (Basel). 2026 May 29;16(6):916. doi: 10.3390/life16060916 (PMC13301241; doi:10.3390/life16060916)
Supplement: Supplementary file 1 [file life-16-00916-s001.zip › life-4315596-supplementary.pdf]

life MDPI

Supplementary Table S1. Preclinical Evidence Grade for MNP induced Myocardial Injury Pathways

Pathway/Endpoint	Evidence Type	Model systems	Consistency of Findings	Overall evidence grade
Oxidative Stress	In vitro · animal · avian · organoid · hiPSC-CM	Fish, rodent, chicken, zebrafish, hiPSC-CM; multiple polymer types (PS, PP, PET, PE)	↑ MDA and ↓ SOD/GSH-Px/CAT replicated across fish, avian, rodent, and hiPSC-CM models, and across multiple polymer types. Size-dependent effect confirmed. Most consistently replicated finding in the MNP cardiac literature.	**Consistent preclinical evidence**
Mitochondrial dysfunction	In vitro · animal · avian	Fish, rodent, chicken; PS-NPs and PET-MPs; ultrastructural confirmation on electron microscopy	↓ MMP, ↓ ATP, cytochrome C release, and mitochondrial fragmentation replicated across fish, avian, and rodent models. Ultrastructural abnormalities confirmed histologically across independent studies.	**Consistent preclinical evidence**
Calcium Dysregulation	In vitro · organoid · animal	hiPSC-CM, cardiac organoid-on-a-chip, murine models; primarily PS-NPs	SERCA inhibition and impaired calcium transients demonstrated in organoid and hiPSC-CM systems. Fewer independent replications than oxidative stress or mitochondrial dysfunction. Bidirectional feedback with mitochondrial dysfunction mechanistically plausible but not fully characterized.	**Moderate preclinical evidence**
ER Stress	In vitro · animal	Murine cardiomyocytes, AC16 human cardiomyocytes; PS-NPs	UPR activation (CHOP, XBP-1) demonstrated but limited independent replication. Cardiac-specific contribution to MNP-induced injury requires further characterization in diverse model systems.	**Moderate preclinical evidence**
Cardiomyocyte Death (apoptosis, pyroptosis, ferroptosis)	In vitro · animal · avian · organoid	Fish, rodent, chicken, human organoids; PS-NPs, PP-MPs, PE-NPs	Apoptosis and pyroptosis: replicated across multiple independent studies and model systems via Bax/BCL2/caspase-9 and NLRP3/caspase-1 respectively. Ferroptosis: emerging evidence with fewer independent replications. Overall consistency is pathway dependent.	**Consistent (apoptosis /pyroptosis) Moderate (ferroptosis)**
Myocardial Fibrosis	In vitro · animal	Rodent models (rat and mouse); PS-MPs and PS-NPs; histological collagen quantification	Collagen I/III deposition confirmed histologically across independent rodent studies. Wnt/β-catenin and TGF-β1/Smad2/3 pathways replicated. No human histological data available.	**Moderate preclinical evidence**

Systolic Dysfunction	Animal · avian	Murine models (C57BL/6, BALB/c, ICR); PS-NPs, PE-NPs, PS-MPs; echocardiographic assessment	Dose- and duration-dependent ↓ EF/FS, ↑ LVIDd, and eccentric remodeling replicated across multiple independent murine studies and polymer types. Transcriptomic enrichment for DCM gene sets at 12 weeks provides molecular corroboration. Experimental doses substantially exceed estimated human blood MNP concentrations; translational relevance to chronic human exposure remains uncertain.	**Moderate preclinical evidence (human translation uncertain)**
Human DCM association	Observational human (tissue detection) · hiPSC-CM	Cardiac surgical patients (myocardium, pericardium); hiPSC-CMs for dose-dependent oxidative stress and apoptosis	MNPs detected in human myocardium and pericardium. hiPSC-CM data show dose-dependent oxidative injury and apoptosis. General cardiovascular associations (MACE) established in observational studies. No prospective data linking MNP exposure to incident DCM. General cardiovascular toxicity should not be conflated with DCM-specific remodeling.	**Limited / indirect evidence (no prospective human DCM data)**

Evidence is graded across six mechanistic pathways and two clinical endpoints based on consistency of replication, model systems used, and availability of human data. All findings are derived from preclinical or observational studies.
